# Identification of Biomarkers Associated With Paget's Disease of Bone and Bone Metastasis From Breast Cancer Patients

**DOI:** 10.1002/cnr2.70003

**Published:** 2024-09-05

**Authors:** Mahima Bhardwaj, Farhana Begum, Duleswar Singh, Srirama Krupanidhi, Virendra Kumar Yadav, Dipak Kumar Sahoo, Ashish Patel, Sachidanand Singh

**Affiliations:** ^1^ Department of Biotechnology, Vignan's Foundation for Science Technology and Research (Deemed to Be University) Guntur Andhra Pradesh India; ^2^ Department of Biotechnology Institute of Life Sciences Bhubaneswar Odisha India; ^3^ Marwadi University Research Center Marwadi University Rajkot Gujarat India; ^4^ Department of Veterinary Clinical Sciences, College of Veterinary Medicine Iowa State University Ames Iowa USA; ^5^ Department of Lifesciences Hemchandracharya North Gujarat University Patan Gujarat India; ^6^ Department of Biotechnology, Faculty of Energy Technology Pandit Deendayal Energy University Gandhinagar Gujarat India

**Keywords:** biomarker, bone metastasis, differentially expressed genes, high‐throughput transcriptome sequencing, Paget's disease of bone

## Abstract

**Background:**

The bone is among the most frequently chosen sites for the metastatic spread of breast cancer. The prediction of biomarkers for BM (Bone Metastasis) and PDB (Paget's disease of bone) initiated from breast cancer could be critically important in categorizing individuals with a higher risk and providing targeted treatment for PDB and BM.

**Aims:**

This research aims to investigate the common key candidate biomarkers that contribute to BM‐BCa (Bone metastasis of breast cancer) and PDB by employing network decomposition and functional enrichment studies.

**Methods and Results:**

This research analyzed high‐throughput transcriptome sequencing (RNA‐Seq). For this work, the dataset (GSE121677) was downloaded from GEO (Gene Expression Omnibus), and DEGs were identified using Galaxy and R script 4.3. Using STRING (Search Tool for the Retrieval of Interacting Genes), high‐throughput research created a protein‐protein interaction network (PPIN). The BM‐PDB‐interactome was created using Cytoscape 3.9.1 and PDB biomarkers, with the top 3% DEGs from BM‐BCa. Functional Enrichment Analysis (Funrich 3.1.3) and DAVID 6.8 performed functional and gene set enrichment analysis (GSEA) of putatively essential biomarkers. TCGA (The Cancer Genome Atlas) validated the discovered genes. Based on our research, we identified 1262 DEGs; among these DEGs, 431 genes were upregulated, and 831 genes were downregulated. During the third growth of the interactome, 20 more genes were pinned to the BM‐PDB interactome. RAC2, PIAS1, EP300, EIF2S1, and LRP6 are among the additional 25% of genes identified to interact with the BM‐PDB interactome. To corroborate the findings of the research presented, additional functional and gene set enrichment analyses have been performed.

**Conclusion:**

Of the five reported genes (RAC2, PIAS1, EP300, EIF2S1, and LRP6), RAC2 was identified to function as the common key potential biomarker in the BM‐PDB interactome analysis and validated by TCGA in the study presented.

## 1 Introduction

Breast cancer (BCa) is the most prevalent cause of mortality from cancer among women worldwide. Women between the ages of 12 and 55 are at high risk of having BCa [1]. It is the most common disease among people, with over 2.3 million cases diagnosed yearly, according to the World Health Organization (WHO). If yearly mortality falls by 2.5% each year, 2.5 million BCa deaths will be prevented between 2020 and 2040 [2]. BCa may arise in any breast area, like the ducts or lobules, and can spread to other body parts via blood and lymph vessels [3, 4]. Patients with BCa frequently develop bone metastasis (BM), and treating them is an essential and challenging element in the metastatic scenario. Pain, pathological fractures, and spinal cord compression are some of the clinical consequences of BM. When cancer metastasizes to the bones, it is usually incurable, making it a devastating event in a patient's life. A significant proportion (70%) of patients with BCa experience osteolytic bone lesions, which contribute significantly to morbidity and mortality. Unfortunately, when BCa cells move to distant organs, traditional medicines (hormone therapy and chemotherapy) frequently fail to cure the illness [5].

A frequent bone ailment called Paget's disease of the bone is characterized by a disordered remodeling of the bone. This condition can cause serious complications such as fissures, fractures, bone deformities, secondary osteoarthritis, deafness, and neurological problems, even though it is often asymptomatic. There is ample evidence of a remarkable decrease in PDB (Paget's disease of bone) mortality, clinical severity, prevalence, and incidence in many nations, especially in former British colonies [6, 7].

A multitude of features of Paget's disease and BM in cancer patients is similar. Both are distinguished by a regional rise in osteoclast (OCL) production that results in the resorption of bone. Common mediators of increased OCL formation include interleukin‐6 (IL‐6) and receptor activator nuclear factor‐kB ligand (RANKL) and the increased osteoclastogenic character of the bone microenvironment in PDB and BM [8]. PDB, like BM, can be highly localized to a single bone or involve numerous bones. Similarly, bone scans can help both kinds of people spot lesions [9].

Biomarkers play a pivotal role in facilitating the timely identification of diseases, as well as assessing the efficacy of therapeutic interventions, among other pertinent applications [10, 11]. The discovery of evaluating connected interactions is becoming more important in the development of biomarkers associated with diseases that have been biologically altered through illness genesis, growth, or treatment. Understanding molecular etiology, risk assessment, illness classification, monitoring, therapeutic response, and toxicity evaluations rely on the relevance and study of biomarkers [12]. Clinical bioinformatics is critical in identifying and validating disease‐specific biomarkers [13]. Thus, it reveals that exploring one of the most frequent and severe bone disorders, PDB, and associating it with the common core indicators of BM from BCa may provide significant key biomarkers. Recent advancements in bioinformatics and network science analysis interpret high‐dimensional biological data using powerful computational methods [14, 15].

The present study documents RNA‐Seq data analysis to determine key biomarkers responsible for the association of BM‐BCa with PDB. Finding biomarkers shared by the two disorders may significantly improve the ability to identify high‐risk individuals and provide more targeted and efficient treatment plans. The study intends to address a significant gap in knowledge and establish a foundation for improved diagnostic and treatment approaches for patients with BCa‐induced BM and PDB by examining these biomarkers using cutting‐edge bioinformatics techniques.

## 2 Materials and Methods

**FIGURE 1:**
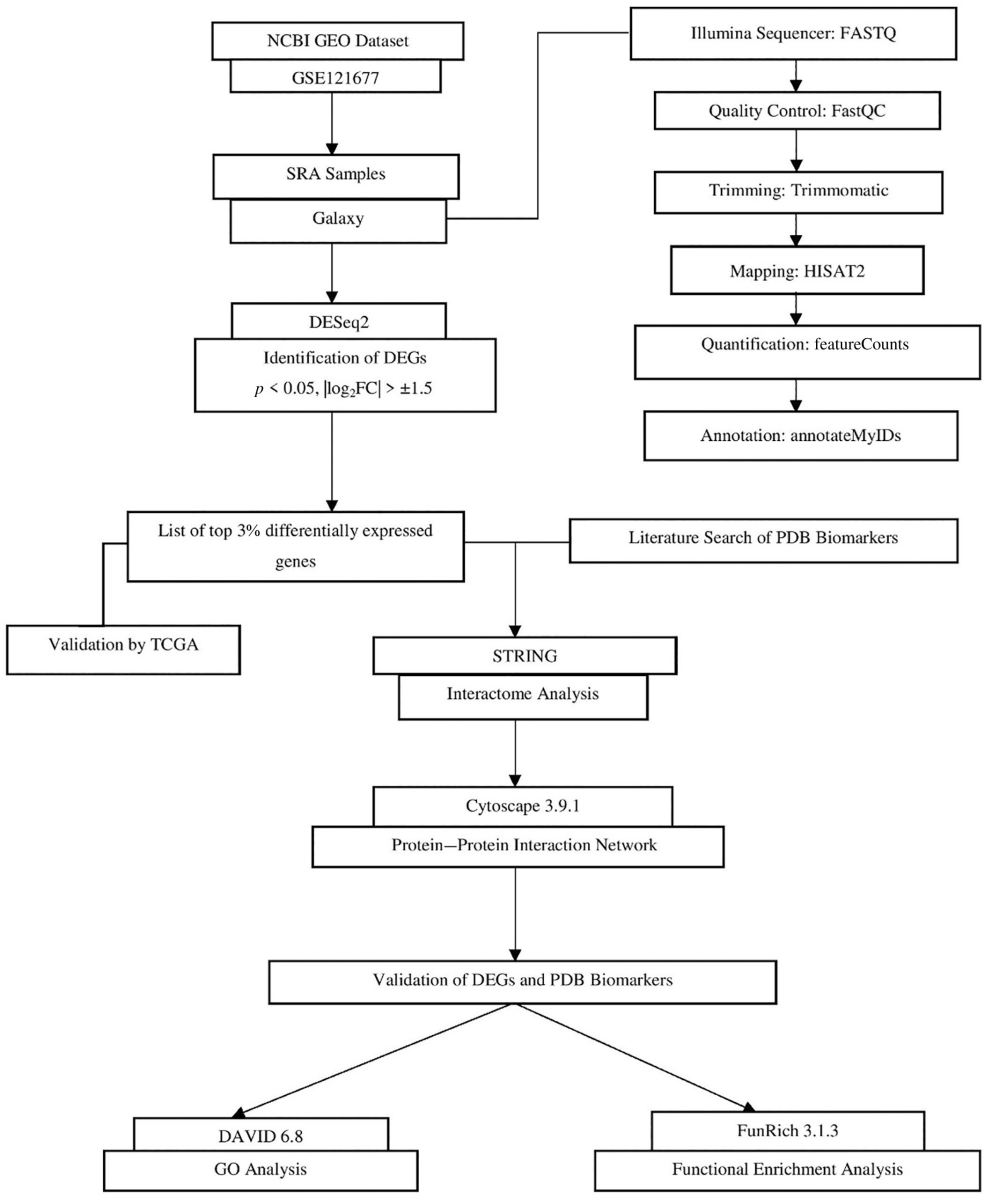
Schematic representation of the present study.

A succession of databases and tools were used to process the raw expression data and predict the outcome. The present study documents RNA‐Seq data analysis to investigate the DEGs (differentially expressed genes) using the Galaxy platform and R script 4.3, and DEGs are validated by TCGA (The Cancer Genome Atlas) program. For PPI (protein–protein interaction) network analysis and to study the role and functional similarity, STRING and Cytoscape 3.9.1 were used. Further, it was investigated for GO (gene ontology) analysis and GSEA (gene set enrichment analysis) using DAVID 6.8 and FunRich 3.1.3 to identify the biological pathways of the significant genes and to determine key biomarkers responsible for the association of BM‐BCa with PDB. Gene expression profiles from the TCGA were used to validate the reported genes. Figure 1 depicts the schematic flow of the presented research.

### 2.1 Data Retrieval

The NCBI GEO database (https://www.ncbi.nlm.nih.gov/geo/) was searched with the phrase “Bone metastasis AND breast cancer” that filters unique to an organism, that is, *Homo sapiens*. The GEO dataset GSE121677 contains information from six samples, three of which are BCa samples and three of which are BM‐BCa samples. The collection was formed from spontaneous metastasis from the mammary fat pad to the bone marrow in the absence of exogenous estrogen. High throughput sequencing for gene expression profiling of GSE121677's RNA expression profile was determined using the GPL18573 Illumina HiSeq 500 (*H. sapiens*). The dataset GSE121677 is a vital source for this research project. DEGs linked to bone metastases of breast cancer (BM‐BCa) can be found in this dataset thanks to high‐throughput transcriptome sequencing (RNA‐Seq) data. RNA‐Seq data from primary BCa and BM were obtained from the SRA database (SRA study PRJNA498077) [16]. The parameters for the dataset GSE121677 retrieved from GEO are listed in Table 1.

**TABLE 1:** Parameters for the NCBI GEO dataset GSE121677.

S. no.	Parameter	Explanation
1	GEO accession number	GSE121677
2	BioProject	PRJNA498077
3	Month and year	Sep, 2021
4	Data type	RNA‐Seq
5	Cell line	MCF7
6	Instrument	NextSeq 500
7	Platform	illumina
8	Source name	Human MCF7 breast cancer cells
9	SRA study	SRP166532
10	SRA sample	12: Primary breast cancer 12: Bone metastatic breast cancer

### 2.2 Data Normalization and Processing

For GSE121677, raw FASTQ files were retrieved and analyzed using the Galaxy web interface platform (https://usegalaxy.org/) to acquire raw counts, as the submitted input was raw RNA‐Seq reads [17, 18]. The raw read's quality was assessed using FASTQC, and a FASTQC HTML report was generated [19]. Second, Trimmomatic was then used to delete the adapter sequences [20]. The adapter‐trimmed reads were aligned to a reference genome with the sequence aligner HISAT2, with all other options set to default [21]. Following alignment, the resulting BAM files were used to create raw counts for each RNA‐Seq data sample using the featureCounts tool (Data S1) [22].

### 2.3 Differential Expression Analysis

The raw counts of each of the SRA sample files (SRR8097383, SRR8097384, SRR8097385, SRR8097386, SRR8097387, SRR8097388, SRR8097389, SRR8097390, SRR8097391, SRR8097392, SRR8097393, SRR8097394, SRR8097395, SRR8097396, SRR8097397, SRR8097398, SRR8097399, SRR8097400, SRR8097401, SRR8097402, SRR8097403, SRR8097404, SRR8097405, and SRR8097406) of the GEO dataset GSE121677 were processed independently as a counts data file and col data file for quality control and differential gene expression analysis was performed by R script using the DESeq2 tool as a computational approach. R version 4.3 was used to annotate raw count data by mapping Entrez Gene IDs and Gene symbols. Gene symbols with no Entrez ID, no counts data, or duplicates were removed as the filter criteria. DEGs were screened based on significant *p*‐value < 0.05 and |log_2_FC| > ±1.5 [23].

### 2.4 Validation by TCGA

TCGA, a project launched by the NCI (National Cancer Institute) in 2005, aims to uncover genetic alterations linked to cancer through comprehensive genome‐wide sequencing and advanced analytics. Over 20 000 primary cancer and matched normal samples covering 33 cancer types have been molecularly characterized by TCGA. The TCGA database comprises plenty of specimens of tissue that are analyzed through a variety of methods, including genomic expression. Furthermore, the data are publicly accessible to all researchers for their particular investigations. Therefore, the present study utilized the extensive TCGA RNA‐Seq dataset. CMI‐MBC (Count Me In: The Metastatic Breast Cancer) project was retrieved to gather data on BCa metastasis [24]. The retrieved RNA‐Seq data contain 200 BCa metastatic samples, including 168 primary tumors and 32 metastasis samples.

### 2.5 Literature Mining of Significant PDB Biomarkers

The present study focuses on the identification of biomarkers in both diseases, as a couple of analogies exist between BM‐BCa and PDB patients. Through literature mining, a total of 16 biomarkers were identified that are evident to contribute to PDB. Those significant genes are TNFRSF11A [25], TNFSF11 [26, 27], TNFRSF11B [28, 29], SPP1 [30], SOST [31, 32], DKK‐1 [33, 34], SFRP‐1 [35], CALCA [33, 34], CEACAM7 [35, 36], CYFRA [35, 37], CXCR4 [38, 39], ACKR3 [39, 40], IBSP [41], SIL1 [30, 42], POSTN [43], and APD [44, 45]. Table 2 represents various literature searches and reporting functions for the significant PDB biomarkers.

**TABLE 2:** List of known biomarkers for the PDB with reported functions.

Biomarkers of PDB	Reported function	References
TNFRSF11A	Participates in the function of osteoclast mediation and analyzes metabolism of bone	[25]
TNFSF11	Acts as a crucial component in the process of differentiation and osteoclast activation, regulation of T cell‐dependent immune response, and stimulates antiapoptotic kinase	
TNFRSF11B	Mediator for lymph node development, osteoclast differentiation, and activity [28, 29]	[26, 27]
SPP1	Involves in the binding of a calcified matrix of bone to osteoclasts. The protein that has been encoded is released and exhibits a strong affinity for hydroxyapatite	[30]
SOST	Loss‐of‐function mutations in this gene cause progressive bone overgrowth	[31, 32]
DKK‐1	Holds a crucial function in embryonic development and adult bone formation	[30, 32]
SFRP‐1	Acts as soluble modulators of Wnt signaling, epigenetic suppression of SFRP genes leads to dysregulated activation of the Wnt pathway, which is linked to cancer	[30]
CALCA	Involves in calcium regulation, phosphorus metabolism, vasodilator, and acts as an antimicrobial peptide	[33, 34]
CEACAM7	Activity of this gene may be reduced in colon and rectal cancers	[35, 36]
CYFRA	Used as a diagnostic biomarker for gastrointestinal cancers, regulates differentiation and polarity of the cell	[35, 37]
CXCR4	It collaborates with the CD4 receptor to aid HIV entrance into the cells and is also abundant in the cancer cells	[39]
ACKR3	Acts as a receptor for vasoactive intestinal peptide (VIP), coreceptor for human immunodeficiency viruses (HIV)	[39, 40]
IBSP	Interacts with calcium and hydroxyapatite through clusters of acidic amino acid and enables attachment of cells via an RGD motif that identifies the vitronectin receptor	[41]
SIL1	Used as a nucleotide exchanging factor for a response from the other unfolded protein	[30, 42]
POSTN	Involves in tissue formation and regrowth, as well as cancer stem cell survival and metastasis	[43]
APD	Acts as regulators of osteoclast development, lymph‐node organogenesis, and vascular calcification	[44, 45]

### 2.6 Construction of the PPI Network

Using a web‐based database called STRING, a PPIN of DEGs was created, which is intended for assessing PPI interactome analysis [46]. Using Cytoscape 3.9.1, an interactome of PDB biomarkers and the top 3% of BM DEGs (the BM‐PDB interactome) was created to scrutinize the PPI network interactive analysis. In addition to Cytoscape, Clusterviz plugin was installed to find multiple clusters in a network. Clusterviz fascinates on the extensively utilized algorithms, namely, MCODE, FAG‐EC, and EAGLE. MCODE's algorithm was used in the present study because it finds extremely connected fragments in an area of the network to build biological networks using correlation analysis, which has three processes: vertex weighting, postprocessing, and complex prediction in filtering or adding proteins in the generated complex. A web composed of interconnected molecules might be visualized graphically, with nodes and edges, respectively. MCODE employs strategies based on the coefficient of clustering to determine a vertex's neighborhood to locate locally dense sections in a network. A clique is defined as the most linked network using all the MCODE options to default [47]. Here, Tables 3 and 4 demonstrate a list of the top 40 up and downregulated DEGs.

**TABLE 3:** List of the top 20 upregulated genes.

GS	BM	Log_2_FC	lfcSE	TS	*p*	*p*_adj_
SPANXN3	10.51	7.12	0.62	11.32	9.96e−30	7.87e−29
SPANXN4	10.23	6.57	0.63	10.30	6.65e−25	4.52e−24
EIF1	59.11	5.95	0.28	20.89	5.50e−97	1.48e−95
SPANXA2‐OT1	3.84	5.39	0.65	8.20	2.36e−16	1.18e−15
LINC00624	3.92	4.84	0.67	7.22	4.86e−13	2.09e−12
H2BC8	33.62	4.45	0.24	18.31	6.64e−75	1.33e−73
AGAP5	3.46	4.43	0.66	6.69	2.22e−11	8.77e−11
VENTX	9.16	4.39	0.44	9.95	2.33e−23	1.51e−22
CRNN	1.82	4.28	0.70	6.09	1.07e−09	3.82e−09
MSMB	38.16	4.24	0.21	19.94	1.77e−88	4.28e−87
SHISA2	78.50	4.16	0.14	28.87	2.33e−183	1.35e−181
ZCCHC14‐DT	1.31	4.14	0.73	5.65	1.54e−08	5.11e−08
SLC52A2	5.66	4.08	0.52	7.81	5.31e−15	2.49e−14
INTS3	6.74	4.07	0.96	4.22	2.34e−05	6.03e−05
BLACAT1	8.74	3.96	0.40	9.79	1.21e−22	7.66e−22
SNORA50A	2.48	3.92	0.67	5.79	6.78e−09	2.31e−08
ARHGDIB	8.47	3.72	0.38	9.69	3.29e−22	2.04e−21
RNASEK	13.57	3.71	0.30	12.15	5.70e−34	5.07e−33
TRGV3	36.00	3.70	0.18	19.85	9.19e−88	2.21e−86
DYDC1	1.76	3.66	0.71	5.16	2.43e−07	7.38e−07

Abbreviations: BM: Base_mean, GS: Gene_symbol, lfcSE: log_2_Fold_changeStandardError, log_2_FC: log_2_Fold_change, *p*_adj_: *p*_adjvalue, TS: Test_stat.

**TABLE 4:** List of top 20 downregulated genes.

GS	BM	Log_2_FC	lfcSE	TS	*p*	*p*_adj_
QRFP	430.50	−10.66	0.58	−18.10	2.97e−73	5.78e−72
SENP6	132.01	−10.18	0.59	−17.25	1.05e−66	1.81e−65
LINC00911	30.93	−8.07	0.59	−13.55	7.30e−42	7.95e−41
NDUFB5	60.05	−7.81	0.59	−13.20	7.80e−40	8.10e−39
TUT4	25.52	−7.80	0.59	−13.12	2.43e−39	2.49e−38
RGS1	34.77	−7.74	0.59	−13.05	6.03e−39	6.13e−38
ARL6IP6	71.53	−7.60	0.54	−13.88	7.99e−44	9.05e−43
PIBF1	58.03	−7.53	0.57	−13.07	4.35e−39	4.44e−38
S100A8	23.32	−7.43	0.59	−12.44	1.42e−35	1.32e−34
STAT1	18.20	−7.28	0.60	−12.06	1.67e−33	1.47e−32
DDX11‐AS1	131.19	−7.22	0.35	−20.51	1.49e−93	3.85e−92
SDAD1	15.70	−7.08	0.59	−11.86	1.79e−32	1.53e−31
CLK2P1	26.5	−6.89	0.59	−11.60	3.69e−31	3.03e−30
FUNDC1	19.48	−6.88	0.59	−11.50	1.25e−30	1.01e−29
SSX1	21.51	−6.82	0.59	−11.46	2.05e−30	1.65e−29
PALMD	14.95	−6.77	0.59	−11.32	9.58e−30	7.58e−29
TMEM68	12.12	−6.70	0.60	−11.04	2.29e−28	1.73e−27
S100A9	81.61	−6.61	0.36	−18.18	6.47e−74	1.27e−72
DBNL	128.14	−6.35	0.26	−23.79	3.57e−125	1.30e−123
PEX3	231.00	−6.35	0.19	−32.02	5.11e−225	3.80e−223

Abbreviations: BM: Base_mean, GS: Gene_symbol, lfcSE: log_2_Fold_changeStandardError, log_2_FC: log_2_Fold_change, *p*_adj_: *p*_adjvalue, TS: Test_stat.

### 2.7 Integrated Enrichment Analysis of Candidate Key Biomarkers

A comprehensive study involving functional and gene set pathway enrichment analysis was conducted in the present study. The identified DEGs were considered for labeling functional annotations and enrichment analysis of the candidate genes by GO analysis [48]. DAVID 6.8, a functional enrichment tool, was used to perform enrichment analysis [49]. The study concentrated primarily on functional annotations of GO, including the ontologies of cellular components (CCs), biological processes (BP), and molecular function (MF). GO terms with (gene count ≥2) were declared statistically significant [50, 51]. The identified DEGs were matched to protein databases and were performed Functional Enrichment Analysis (Funrich 3.1.3) [52]. Moreover, on the other hand, the GSEA was also done to identify significant biological pathways shown by RNA‐Seq [51, 52].

## 3 Results

### 3.1 Identification and Screening of DEGs

**FIGURE 2:**
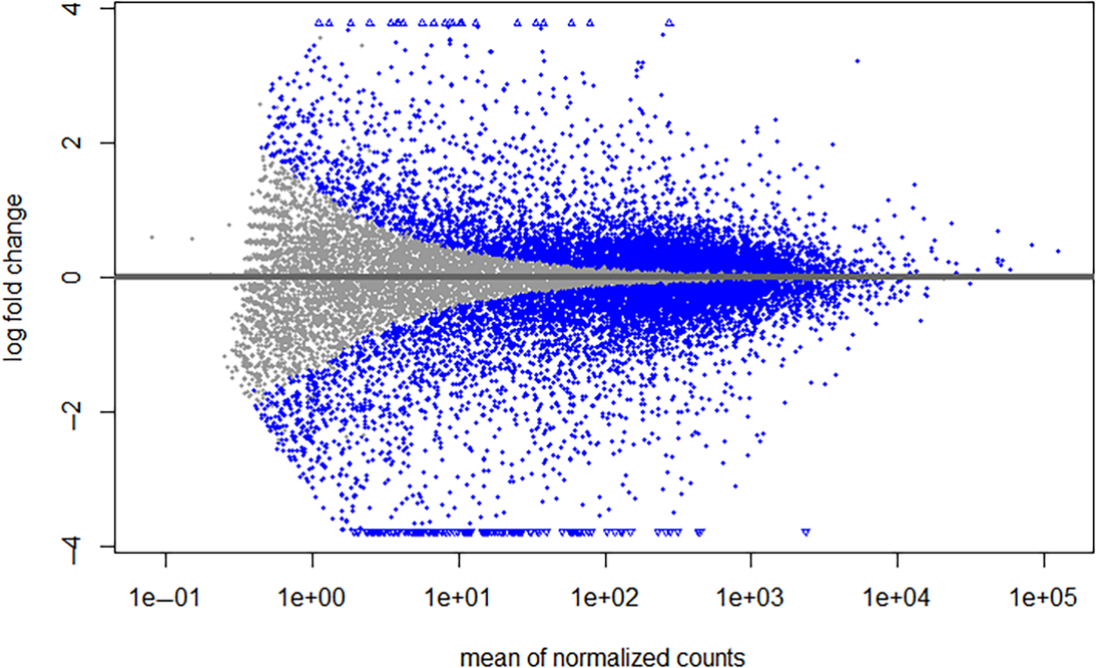
MA (moving average) plot constructed using threshold as |log_2_FC| > ±1.5, for reported DEGs.

The 24 SRA samples were obtained from the NCBI GEO Dataset GSE121677, which included approximately 12 primary BCa samples and the remaining 12 bone metastatic BCa samples processed through the Galaxy platform. FASTQC analyzed the raw data quality, and an HTML report was generated. The adapter sequences were subsequently deleted using Trimmomatic. The adapter‐trimmed reads were aligned to the HISAT2 sequence aligner. The generated BAM files were then used to create raw counts for each RNA‐Seq data sample using the tool named featureCounts. By analyzing the RNA‐Seq read counts of the various genes with R script 4.3 using the computational approach and then DEGs were screened with the significant cut‐off criteria: *p*‐value < 0.05 and |log_2_FC| > ±1.5, and we identified 1262 genes as DEGs from the GSE121677 dataset, including 431 upregulated and 831 downregulated genes. The total 1262 genes based on the significant cut‐off criteria are provided in Data S2, and the MA (moving average) plot is shown in Figure 2.

### 3.2 TCGA Validation

The TCGA gene expression databases were utilized for determining the information about gene expression in the study presented. The retrieval of RNA‐Seq data, which contains 200 BCa metastatic samples, including 168 primary tumor samples and 32 metastasis samples, has been considered to yield 2550 DEGs according to the specified criteria *p*‐value < 0.05 and |log_2_FC| > ±1.5. The results have been linked to the Human GRCh38/hg38 chromosomal standardized reference. Of the top 40 DEGs chosen, 47% are considered to be highly expressed by TCGA.

### 3.3 Network Analysis: BM‐PDB Interactome

**FIGURE 3:**
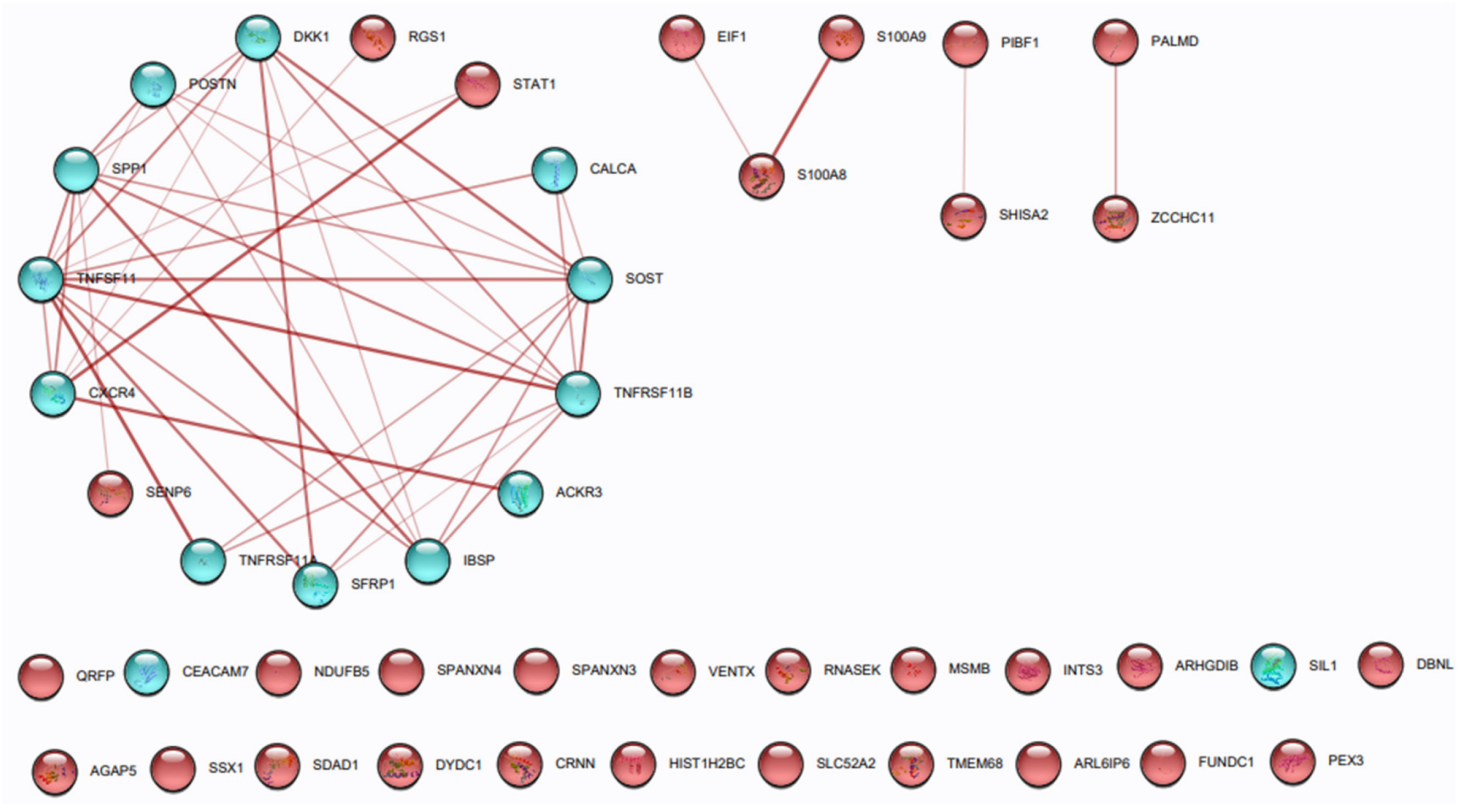
BM‐PDB interactome network (red color nodes: DEGs and blue color nodes: PDB biomarkers) constructed by STRING to observe the interactions between PDB and BM‐related DEGs.

**FIGURE 4:**
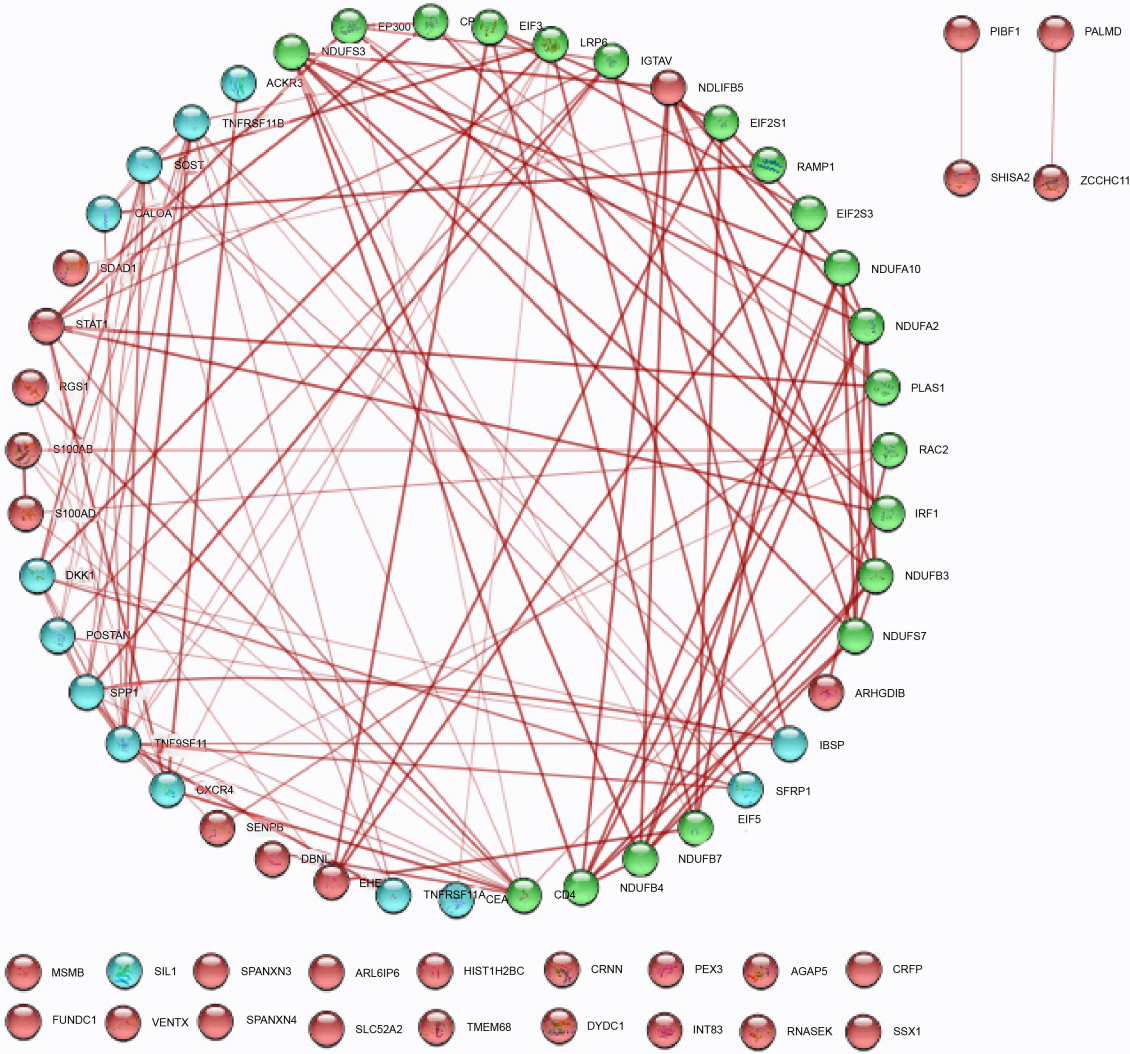
Network extension of the BM‐PDB interactome with the added genes (red color nodes: up and downregulated DEGs, blue color nodes: PDB biomarkers, and green color nodes: added genes).

**FIGURE 5:**
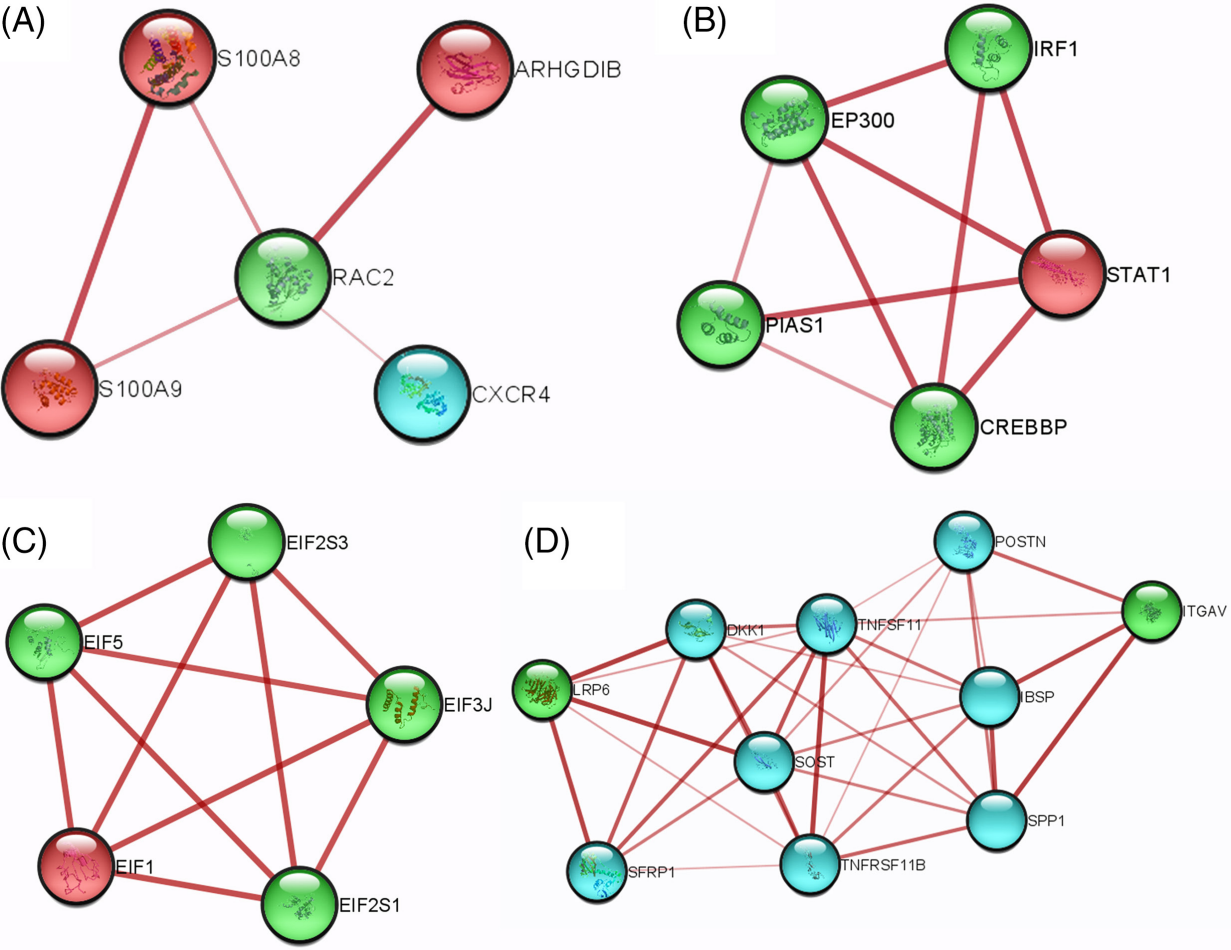
Four clusters (A, B, C, and D) developed by ClusterViz for the distinct BM‐PDB interactome. Here, red color nodes are DEGs, blue color nodes are PDB biomarkers, and green color nodes are added genes.

The PPIN of DEGs, containing BM‐PDB interactome network of which 45 and 42 are nodes and edges, respectively, of the analyzed RNA‐Seq data was submitted to STRING (Figure 3). Due to 23 seed genes exhibiting direct interactions, gene interactions were noted using Cytoscape 3.9.1 until the third growth. As of the third growth, the enlarged PPI network has 65 nodes and 121 edges, in which Cytoscape 3.9.1 was used to differentiate between the proposed 23 seed genes (DEGs and PDB biomarkers) and the 20 additional genes introduced up to the third growth (Figure 4). For further GO analysis, these five (RAC2, PIAS1, EP300, EIF2S1, and LRP6) of the 20 additional genes were chosen as these genes were observed to be highly connected with DEGs of BM‐BCa and PDB in BM‐PDB interactome. The clusters (A, B, C, and D) formed were based on the plugin ClusterViz of the candidate key biomarkers of the BM‐PDB interactome (Figure 5).

### 3.4 Integrated Enrichment Analysis of Candidate Key Biomarkers

The integrated enrichment analysis, which includes functional and biological pathway enrichment analyses, was carried out. DAVID 6.8 and FunRich 3.1.3 were used to perform functional enrichment analysis, including GSEA, to obtain further insight into the function of identified genes. Separate enrichment studies of the five genes were carried out (Data S3 and S4).

#### 3.4.1 Functional Enrichment Analysis

**FIGURE 6:**
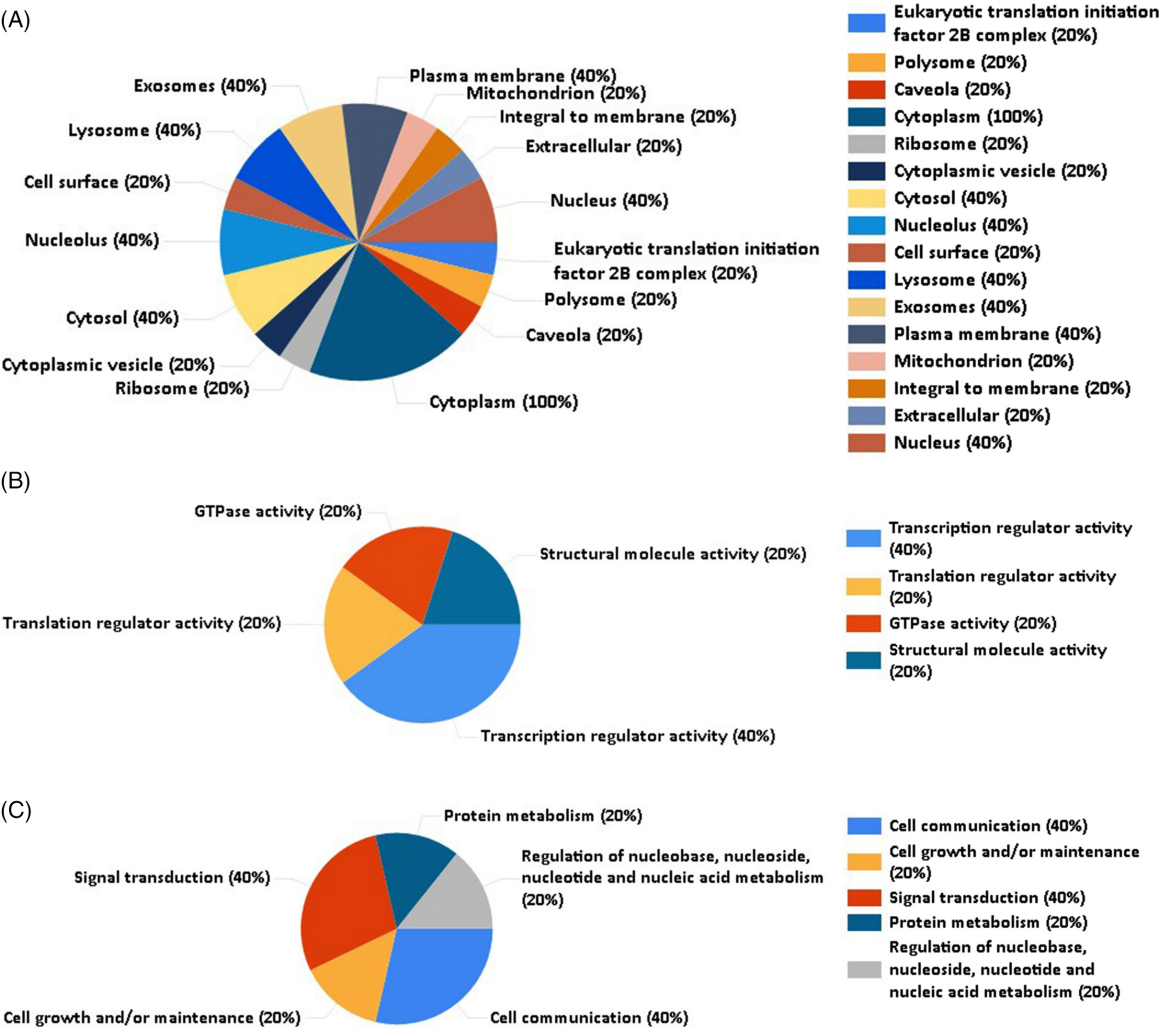
Three pie charts (A, B, and C) developed by FunRich 3.1.3 for the gene ontology analysis. Here, (A) cellular component for gene ontology, (B) molecular function for gene ontology, and (C) biological process for gene ontology.

The gene abundance was primarily focused on the GO analysis and the biological terms (MF, BP, and CC). In CC, the top percentage of genes were enriched in the cytoplasm, that is, 100% (Figure 6A). In MF, the top percentage of genes were enriched in transcription regulator activity, which is 40% (Figure 6B). In contrast, in BP, the top percentage of genes were enriched in cell communication and signal transduction, which is 40% (Figure 6C).

#### 3.4.2 Biological Pathway Enrichment Analysis

The biological pathway enrichment analysis was carried out in order to identify the biological pathways of the five key RNA‐Seq candidate vital biomarkers. Table 5 describes the details of the GO and biological pathway functions of five distinct key candidate genes.

**TABLE 5:** Gene Ontology: Biological pathways and functions of five distinct key candidate genes.

Gene name	Molecular function	Biological process	Cellular component	Biological pathway
RAC2	GTPase activity	Cell communication, signal transduction	Plasma membrane, cytosol, cytoplasm, exosomes, and lysosomes	Wnt signaling pathway [53], pathways in cancer [54], adherens junction [55]
PIAS1	Transcription regulator activity	Cell communication, signal transduction	Nucleus, cytoplasm	Hepatitis C [56], JAK–STAT signaling pathway [57]
EP300	Transcription regulator activity	Regulation of nucleobase, nucleoside, nucleotide, and nucleic acid metabolism	Nucleus, nucleolus, cytoplasm	Wnt signaling pathway, pathways in cancer, adherens junction, JAK–STAT signaling pathway, Influenza A [58]
EIF2S1	Translation regulator activity	Protein metabolism	Nucleus, nucleolus, mitochondrion, cytosol, ribosome, cytoplasm, exosomes, lysosome, Eukaryotic Translation Initiation Factor 2B Complex, Polysome	Hepatitis C, Influenza A
LRP6	Structural molecule activity	Cell growth and/or maintenance	Plasma membrane, integral to membrane, cytoplasm, cell surface, extracellular, caveola, cytoplasmic vesicle	Wnt signaling pathway, pathways in cancer

## 4 Discussion

This present research reports an extensive study into the efficacy of interactome investigations to predict clinical outcomes for early prognosis, therapy, and therapeutic response to a specific disease utilizing candidate key biomarkers [59]. Network science provides a framework for studying the biological and molecular mechanisms underlying human diseases; here, network studies are conducted for interactive analysis in BM‐BCa with the PDB. RNA‐Seq technology has recently been widely employed to uncover molecular targets for a particular disease [59, 60]. The utilization of identical key biomarkers for PDB in the presented work plays a significant role in BM‐PDB interactome analysis [60].

The five potential biomarkers that have been identified were subjected to a comprehensive literature survey in order to elucidate their association with PDB and BM. According to the analysis, RAC2, the reported gene that encodes a small guanosine triphosphate (GTP)‐metabolizing protein from the Ras superfamily, is involved in signal transduction in a variety of cellular activities such as chemotaxis, cytoskeletal rearrangement, cellular differentiation, and proliferation. It was also found to have a unique genetic relationship to Crohn's disease [61]. The gene RAC2 with node score of 2.0 was observed to be in first cluster in ClusterViz results where RAC2 shows direct interactions with S100A9 (downregulated DEG with fold change: −6.61), S100A8 (downregulated DEG with fold change: −7.43), ARHGDIB (upregulated DEG with fold change: 3.72), and CXCR4 (PDB biomarker), all these four genes were reported as DEGs in TCGA validation. All these genes interacting with RAC2 have been observed in a common pathway, that is, Wnt signaling pathway and pathways of cancer, when performed GO.

PIAS1, the second reported gene, encodes a protein inhibitor of activated STAT (PIAS) members. By directing the sumoylation of target proteins, PIAS1 proteins act as SUMO E3 ligases and play crucial roles in numerous physiological processes [62]. EP300, another reported gene, encodes the transcriptional coactivator p300 protein associated with adenovirus E1A [63]. Both genes PIAS1 and EP300 were present in cluster 2, constructed by ClusterViz, with node scores of 3.0 and 2.7, respectively. Gene PIAS1 and EP300 show direct interactions with two added genes (IRF1 and CREBBP) and one downregulated gene, STAT1, with fold change: −7.28. Also, it is reported that all the genes of cluster 2 are involved in the JAK–STAT signaling pathway in KEGG.

EIF2S1, another reported gene, promotes the first controlled stage in the start of protein synthesis, encouraging initiator tRNA binding to subunits of 40S ribosomes. This gene has also been linked to neurodegenerative disorders [64]. The gene EIF2S1 with node score of 4.0 was observed to be in the third cluster in ClusterViz results where EIF2S1 shows direct interactions with EIF1 (upregulated DEG with fold change: 5.95), three added genes EIF5, EIF2S3, and EIF3J. All these genes observed in cluster 3 have been reported in a common KEGG pathway, that is, RNA transport.

LRP6 is the fifth reported gene that encodes for low‐density lipoprotein (LDL) receptors. This gene has also been associated with coronary artery disease [65]. The gene LRP6, with a node score of 5.0, was observed to be in the fourth cluster in ClusterViz results, where LRP6 shows direct interactions with PDB biomarkers and one added gene, whereas it shows indirect interactions with DEGs of BM. All these genes interacting with the LRP6 have been observed in the Wnt signaling pathway when performed GO.

As demonstrated in our research, all the reported genes show direct or indirect interactions with BM‐PDB. Of the five reported genes, the first cluster of the RAC2 gene plays a distinct role in the BM‐PDB interactome analysis as it is the only gene that shows direct interaction with the DEGs and PDB biomarkers and is also validated by TCGA.

The key benefit of the present study is that it makes use of the BM‐PDB interactome to build and study the network clusters of five distinct key biomarkers. The interactions between PPI networks are derived in this work for the investigation of one of the most prevalent and severe bone illnesses, PDB, and linking them with the usual core signs of BM‐BCa may give substantial biomarkers [66]. The biomarkers revealed in the study will provide a dynamic and powerful method for comprehending the BM‐PDB illness spectrum, with applications in randomized clinical trials, screening, diagnosis, and prognosis [67]. Biomarkers can also represent the complete disease spectrum, from early signs to late stages. Our findings might be used in future studies to establish gene expression‐based prediction models that may also benefit from their use in clinical practice [68].

## 5 Conclusion

The NCBI GEO Dataset GSE121677, which depicts BM‐BCa, was used in this research to identify common potential biomarkers for PDB and BM‐BCa. The current study detected 431 and 831 up and downregulated DEGs from the retrieved dataset. Out of them, the top 3% DEGs with 16 significant PDB biomarkers based on literature mining were then submitted to STRING and Cytoscape 3.9.1 to be further studied for PPI interactome analysis, which identified the common five distinct candidate key biomarkers until the third growth due to the main seed genes showing direct interactions. These five (RAC2, PIAS1, EP300, EIF2S1, and LRP6) reported genes were chosen for further GO and GSEA analysis and were most abundant for functional enrichment analysis in “cytoplasm, transcription regulator activity, cell communication, and signal transduction” whereas, in pathway enrichment analysis, they were prevalent in “Wnt signaling pathway, pathways in cancer, adherens junction, Hepatitis C, JAK–STAT signaling pathway, Influenza A.” All five reported genes were utilized in ClusterViz, which illustrates the clusters of the five distinct putative key biomarkers along with their first neighbors. Of these, the RAC2 gene acts as a key candidate biomarker that shows direct interaction with the DEGs and PDB biomarkers and is also validated by TCGA. Thus, this finalized reported gene might lead to new insights into molecular disease pathways, paving the way for the creation of reasonable personalized treatment approaches to develop gene expression prediction and prognosis models of a particular disease. It is necessary to validate the presented biomarkers through in vitro and in vivo research, which can potentially be conducted in subsequent studies.

## Author Contributions

**Mahima Bhardwaj:** investigation, writing – original draft, methodology, validation, visualization, writing – review and editing, formal analysis. **Farhana Begum:** investigation, writing – original draft, methodology, validation, visualization, writing – review and editing, formal analysis. **Duleswar Singh:** investigation, validation, methodology, formal analysis, writing – review and editing. **Srirama Krupanidhi:** investigation, methodology, validation, formal analysis, writing – review and editing. **Virendra Kumar Yadav:** investigation, writing – review and editing, validation, formal analysis, supervision, writing – original draft. **Dipak Kumar Sahoo:** writing – review and editing, validation, formal analysis, methodology, conceptualization, writing – original draft. **Ashish Patel:** conceptualization, writing – review and editing, funding acquisition, methodology, formal analysis, validation, supervision, resources, writing – original draft. **Sachidanand Singh:** supervision, resources, data curation, project administration, formal analysis, software, writing – review and editing, visualization, validation, methodology, writing – original draft, investigation, funding acquisition, conceptualization.

## Conflicts of Interest

The authors declare no conflicts of interest.

## Supporting information


Data S1.

Data S2.

Data S3.

Data S4.

## Data Availability

The data that supports the findings of this study are available in the supplementary files and also provided by the corresponding author upon reasonable request.
